# Review of Black Soldier Fly (*Hermetia illucens*) as Animal Feed and Human Food

**DOI:** 10.3390/foods6100091

**Published:** 2017-10-18

**Authors:** Yu-Shiang Wang, Matan Shelomi

**Affiliations:** Department of Entomology, National Taiwan University, Taipei 10617, Taiwan; b02612007@ntu.edu.tw

**Keywords:** black soldier fly, entomophagy, industrial ecology, sustainability, *Hermetia illucens*

## Abstract

Food futurists accept that sustainability-minded humanity will increasingly incorporate insects as alternative protein. The most studied and easily reared species are not necessarily the most sustainable, acceptable, or delicious. Here, we review the literature on the black soldier fly, *Hermetia illucens,* which is capable of efficiently converting a wide variety of organic materials, from food waste to manure, into insect biomass. They can be grown and harvested without dedicated facilities and are not pestiferous. Their larvae are 42% crude protein and 29% fat, although they are higher in saturated fats than most insects. They do not concentrate pesticides or mycotoxins. They are already grown and recommended for use as animal feed, but with regional legal restrictions on how this is done. For commercial use in human foods, larvae could potentially be milled and converted into a textured protein with a strong flavor. Their biggest advantage over other insects is their ability to convert waste into food, generating value and closing nutrient loops as they reduce pollution and costs. This general advantage is also their greatest disadvantage, for the social stigmas and legal prohibitions against eating organisms that eat waste are added to extant taboos facing insect consumption.

## 1. Introduction

Entomophagy, meaning consumption of insects, has been practiced by humans on every inhabited continent, not only historically [1,2,3], but also up until the present day [4,5,6,7]. Insects play a more prominent role in the cuisines of some contemporary cultures [8], while in others they are seen as taboo or at least unappetizing [9]. Over the past century, however, starting perhaps with Holt’s [10] essay, “Why Not Eat Insects?”, discussion of the role insects should play in modern, urban, and/or Western food culture has increased [11]. Consumers without longstanding traditions of entomophagy are displaying a growing awareness that insects are edible and have benefits over other foods, and are showing greater interest in trying edible insect dishes [12,13,14,15], either out of curiosity [16], ecological concerns [17], or other reasons [18]. Interest in entomophagy has reached a point where the supply of insects cannot keep pace with the demand [13,19], and some edible species are already under threat of overexploitation [6]. International conferences have been held [6,20,21,22] and cookbooks published on the subject [23,24,25,26]. Particular attention has been paid to the potential of edible insects as a solution to present or looming food crises [27,28,29], in particular fears of global food insecurity due to climate change and/or rising populations [30,31].

Besides being delicious when prepared properly, insects are rich sources of proteins, good fats, and certain trace elements. Their greatest advantage over other animal meats, which underlies their frequent championing as saviors in a food-insecure world, is their lower environmental impact. Insects have a lower feed-to-protein conversion ratio than cattle or swine [32] and even poultry according to some sources [31], and produce fewer greenhouse gases and lower ammonia emissions than any conventional livestock [31,33,34]. Industrial-scale insect farms need less water and land space than pasture [35], can have a lower water footprint per gram of protein than any conventional livestock or even milk and eggs [36,37], and some insect species can even consume organic waste and side-streams [29,31]. Thus insect rearing can operate in developing countries that need low-tech and low-capital investment, yet can still be done with high technology and automated methods to produce consistent, safe, high-quality products [20]. Insects can also improve the environmental footprint of vertebrate meats indirectly, through their use as feed [38,39]. Rearing insects on human-inedible wastes and feeding them to larger food animals (whose wastes can even be fed to the insects in a partly closed circle of food energy) can boost the protein content of these animals [40] and is more environmentally friendly and efficient than growing fields of grains or other feeds, which use land and resources that could otherwise be used to grow food for humans [31].

One known problem with industrializing edible insects today, however, is the relative dearth of insects to choose from. While thousands of species are consumed worldwide [29], all but a dozen or so are wild caught by more traditional societies and cannot at this time be farmed, with consequences for regular supply and for conservation [41,42]. The species commonly sold and consumed in the West, such as house crickets and mealworms, are thus not necessarily the most sustainable species nor those with the most desirable organoleptic properties such as taste and texture. Several authors have noted that, if done improperly, entomophagy can be environmentally destructive rather than helpful [27,41]. Insects that are difficult to rear or harvest and thus in lower supply would also be more expensive, lowering their desirability among most consumers; and of course the issue of flavor in food acceptance cannot be understated. Rearing insects on otherwise inedible organic wastes would greatly lower their environmental footprint and boost their utility, in particular for developing world consumers. An added benefit is the recycling of the waste itself, as the management of organic wastes such as manure, leachates, and food waste is both costly and a growing environmental concern [43]. Rearing edible insects on wastes would solve two problems at once [44], but popular species like cricket and mealworm cannot be reared easily on most waste, especially animal products. A need exists, therefore, to identify and refocus attention on species with superior cultivation properties than extant edible insect species, but which can still be used either as feed or food.

The black soldier fly, *Hermetia illucens*, is a true fly (Diptera) of the family Stratiomyidae. Though originally native to the Americas, it now occurs worldwide in tropical and temperate regions [45,46], and its lack of hardiness to the cold precludes its invasion of nonnative regions such as Northern Europe [47]. Adults consume nothing but water, do not approach humans, do not bite or sting, and do not vector or disseminate any specific diseases [46,48]. Black soldier fly larvae (BSFL) are reported as feeding on an immense variety of organic material, and have already been used in small-scale waste management purposes using substrates such as manure [49,50], rice straw [51], food waste [52], distillers’ grains [53], fecal sludge [54,55], animal offal, kitchen waste, and so on [56]. The diversity of substrates they can process and the efficiency with which they do so may be the highest among the flies [57]. BSFL are also edible, and have been studied as such. Their feed conversion ratios are known to be superior to both crickets and mealworms, and, compared to those two, BSFL survival rate and nitrogen and phosphorus compositions do not vary as highly with diet [32]. They are not thought to be toxic [58]. BSFL accumulate lipids from their diet for use as energy by the non-feeding adult, to the point that they can be converted to biodiesel [59,60,61,62]. What they do not consume, combined with their nitrogen-rich frass, can be used as fertilizer [52,63]. Their larval development time of over three weeks is longer than that of flies such as house and carrion flies (<5 days), meaning a single larva will consume a larger amount of substrate and produce larger pupae [46]. Additionally, when BSFL are at the pre-pupa stage, they will instinctively leave the substrate and move to a high, clean place, a behavior called “self-harvesting” that removes an otherwise labor-intensive step from their farming [45,64]. All these benefits make BSFL practical to rear and a suitable tool to valorize wastes, plus possibly a sustainable animal feed or human food source. Here we review the literature on the black soldier fly to evaluate its suitability for use in human food systems.

## 2. Review

### 2.1. Black Soldier Fly as Livestock Feed

BSFL meal and oil are already considered to be an animal-grade alternative to fish meal and fish oil used to feed carnivorous fish and in other animal diets, due to their high protein and lipid contents even when fed plant-based waste streams [65]. The importance of fish meal and oil in aquaculture is well known, but competition with demands for fish for human consumption and depleted fisheries, among other factors, have brought the supplies of fish meal and oil down and costs up, leading fisheries to search for alternatives such as vegetable oils [66]. BSFL can accumulate lipids in their bodies if fed an appropriately lipid-rich diet, and are generally more palatable to the fish than vegetable oils. Omega-3 fatty-acid-enhanced pre-pupae are produced when the larval diet is supplemented with fish offal [67]. Such “enriched” pre-pupae are suitable fish foods, producing no significant differences in fish growth and vision development when compared to normal fish meal for feeding the rainbow trout, *Oncorhynchus mykiss* [68]. Sensory analysis of trout fillets found no differences among fish fed fish meal, BSFL, or enriched BSFL diets [68]. Another case in rainbow trout recommended defatted BSFL supplementation in the diet of up to 40% without any negative effects on fish physiology or fillet physical quality, but noted a decrease in desirable polyunsaturated fats [69]. Another study on rainbow trout placed the limit at 15% BSFL in the diet for unaffected fish growth [70]. A study on juvenile Jian carp (*Cyprinus carpio* var. Jian) found no difference between BSFL oil and soybean oil on growth performance, but decreased carp lipid deposition as the proportion of BSFL oil in the diet increased [66]. In the case of the turbot, *Psetta maxima*, although BSFL meals had relatively low palatability and nutritive value, the use of BSFL was still recommended as a feasible, partial replacement for fish meal because it was reared on local greenhouse wastes [65]. Experiments with African catfish, *Clarias gariepinus,* found total BSFL substitution of fish meal in diets (where it made up only 25%) had no effect in terms of growth rate and nutrient utilization indices, so BSFL were recommended as an alternative due to their lower cost [71]. Ultimately BSFL’s ability to efficiently produce protein-rich edible biomass from potentially protein-poor organic wastes has led many authors to conclude that BSFL can contribute meaningfully to sustainable aquaculture as partial or total meal replacement [72,73,74], including for aquatic invertebrates such as shrimp [75].

BSFL has also been used in poultry feed as a partial replacement for maize or soy-based feeds, mainly because the species naturally colonizes and breaks down poultry manure and populations are often kept by poultry farms for the purpose of waste management and pollution reduction [76,77]. In experiments with broiler quails, *Coturnix coturnix japonica*, no difference was found between control and two proportions of BSFL meal on productive performance, breast meat weight, and yield [78]. BSFL supplementation had no effect on breast meat sensory aspects and flavor perceptions, oxidative status, or cholesterol composition; and it improved the amino acid contents of the meat towards improved nutritional value (increased aspartic acid, glutamic acid, alanine, serine, tyrosine, and threonine). However, it increased levels of the less-desirable saturated and monounsaturated fatty acids [79]. Similar effects were found with BSFL supplementation in the diet of broiler chickens, *Gallus gallus domesticus*, with the note that using defatted BSFL reduced the negative impact on fatty acid profiles. In both cases the authors found BSFL to be a promising protein source for poultry feed [80], with the authors concluding that BSFL “inclusion guaranteed satisfactory productive performances, carcass traits and overall meat quality.” [81] BSFL supplementation (50%) or total replacement of soybean cake in the diets of laying hens had no impact on hen health or performance and little to no effect on the eggs themselves [82]. BSFL are also highly palatable to poultry, with laying hens reported to seek out BSFL from feeders rather than continue to eat ad libidum provisioned wheat–soy feeds [83]. Thus BSFL are a potential partial substitution for poultry feed, providing added protein with the bonus that BSFL can be reared on the manure of the same birds that consume them, simultaneously valorizing and recycling the waste.

Evolutionarily between fish and poultry are reptiles such as the American alligator (*Alligator mississippiensis*), whose insectivorous young can survive on a diet of dried BSFL but reach smaller sizes on this diet than on the control. The low palatability of unprocessed BSFL (reared in this study on restaurant waste) was blamed, so BSFL are only recommended as a partial alternative for commercial alligator feed [84].

Black soldier flies are also known to reduce the mass and nutrient content of swine manure [56] at efficiencies similar to poultry manure [85,86], with benefits for improved farm hygiene, reduced pest fly populations, and reduced nutrient pollution in runoff [87]. Although the flies would not be produced in sufficient volumes to feed the swine, they can be redirected to other uses such as fish feed [88], and the remaining manure residue used for horticulture, enabling plants to grow in otherwise low-quality soils or even sand [89]. BSFL can be reared on dairy cow manure, which is often mixed with other materials to improve larval yields and total waste reduction due to the high crude fiber content of pure dairy manure that the flies otherwise cannot fully digest [57,90,91]. BSFL can also be reared on slaughterhouse blood and offal, again valorizing wastes from human food production [88].

It is therefore well established that BSFL can be used to feed many vertebrates [92] and can use various vertebrate wastes as a substrate, with no effects on the palatability of the BSFL-fed meats for humans and with significant implications for sustainable and lower-input agriculture in the developing world [64,93]. While the potential benefits are greatest in these developing nations, BSFL and other insect feeds are expected to play larger roles over time in advanced economies such as the United States, due to pledges to reduce waste among food conglomerates seeking approval from increasingly environmentally-conscious consumers and regulators, combined with the volatile prices of fish meal and other feed directing producers to seek alternatives [94].

### 2.2. Nutritional Aspects of Black Soldier Fly Larvae

A repeated concern from fish and poultry studies is that BSFL supplementation of diet often negatively changed the fatty acid profiles of the resulting meat products, decreasing polyunsaturated fats and/or increasing saturated and monounsaturated fats [66,81]. This problem is not unique to BSFL, as beetle larvae are also high in monounsaturated fats [95], but BSFL still have far lower polyunsaturated fatty acid percentages than insects such as housefly maggots, mealworms, and adult crickets [39]. In BSFL this issue might be overcome by varying the fatty acids in their substrates [96], such as by using fish offal [67]. Requiring certain substrates to be used would minimize the advantage BSFL have over other insects in its incredible dietary breadth. Also, one study using restaurant, vegetable processing, and biogas fermentation waste found that neither affected the fatty acid profiles of the resulting BSFL meal significantly [97]. Note that the already-established-as-edible crickets are relatively high in desirable polyunsaturated fats [95,98], although the exact fatty acid profiles of crickets can also be altered by changing the oils in their diet [99,100].

The protein and fat compositions of BSFL are impacted by what they consume (Table 1) [45,67,72,89,97]. The effects are not always linear: an experiment using food waste mixes with known protein/fat compositions found that using more protein resulted in more proteinaceous BSFL, but the percentage of fat in the substrate did not correlate with the larval fat percentages [32]. Also, many sources of variation exist, even in the experimental designs. The methods used to extract proteins from BSFL meals, whether the studies used specifically prepupae or all larvae [101], and whether they performed chitin correction, can all influence the results of the percentages given [102,103], with chitin correction reducing the reported percent protein content by 2–5% [97]. The natural variation among individuals and batches can be significant: commercially available BSFL (sold as animal or pet feed) from the same company (Hermetia GbR, Baruth, Germany) had values ranging from 31.7% to 47.6% crude protein and 11.8–34.3% fat in different studies [65,102,104]. Different strains from around the world also process the same substrates differently, at least in terms of development time and phenotypic plasticity [85].

Despite this diversity, all values consistently point to BSFL as a good source of proteins and lipids, averaging 40.8 ± 3.8% protein and 28.6 ± 8.6% fat (Table 1), both for reared and wild specimens [93]. The findings cement BSFL’s position among other insects as a possible alternative protein source in the scenarios envisioned by entomophagy advocates, such as climate change- or overpopulation-induced food insecurity and space travel. In every case, nutrients were concentrated from the substrate, which always had less fat and protein than the insects [97]. The percent protein of BSFL meal, either from prepupae only or from all larvae, is comparable to that of other insect and plant-based meals, albeit on the low end among insects, and the crude fat percentage is higher than most insect meals and even higher than fish and soy meal [39,72]. When defatted, BSFL meal can have crude protein levels over 60% [97], comparable to other insect meals [39], and will have lower lipid percentages, assuaging concerns over the saturated fat content. Because the defatting step requires processing—which has a financial, environmental, and logistical cost, such as requiring extra machinery or facilities as well as chemical solvents such as hexane, as well as time for the processing steps [105]—it would likely not be used in household rearing or famine-aid scenarios. However, the technology required for partial defatting can be as simple as a mechanical pressing of the larvae prior to milling, and is not out of reach for developing world economies [106].

The micronutrient profiles also depended on the substrate fed. BSFL accumulate calcium (the most abundant mineral in BSFL) and manganese, but do not accumulate sodium or sulfur [97]. BSFL meal has over one order of magnitude more calcium than most other insects (6.6–9.3% by dry weight compared to less than 1% for other insects) [39,97,107] and more than fish meal [39], providing a considerable advantage to BSFL over other insects nutritionally. They also provide adequate levels of other essential minerals and vitamins at a level equivalent or superior to other insects [107]. A nutraceutical benefit was also reported, in that BSFL are rich in C12:0 medium-chain fatty acids [97], which have demonstrated prebiotic effects on the microbiota of livestock [108] and antibiotic effects on gastrointestinal disease-causing bacteria [109]. BSFL can thus be considered an alternative to the increasingly banned use of in-feed antibiotics; however, this would come at the cost of increased lipids in the BSFL meal, which, as already mentioned, comes with nutritionally unfavorable fatty acid profiles [97].

### 2.3. Microbial and Chemical Contaminants

As most research has focused on BSFL development and consumption issues, we know little about their safety or security as a food. While few insects appear to be harmful when eaten [58,110], allergens aside [111], one should not ignore issues such as microbial contamination of insects, both of their gut from their food and of their processed products during storage [112,113,114]. BSFL have been noted to reduce the microbial load of substrates, with processed composts showing lower concentrations of bacteriophages and bacteria such as *Salmonella enteritidis* and *Enterococcus coli* [54,63,115]. However, the larvae themselves can become contaminated with these bacteria if kept on contaminated substrate for too long [116]. Data on other, plant-feeding insects suggests *Enterobacteriaceae* are a problem with raw insects [114] and *Clostridium botulinum* causing fatalities in poorly-stored termites in Kenya [117]. Decontamination of harvested BSFL, especially those reared in manure or human solid waste, needs to be incorporated into any plan for an industrial-scale BSFL production facility. BSFL may also have companion bacteria that promote their growth and development: a study found that adding strains of *Bacillus subtilis* isolated from BSFL to poultry manure increased the growth and development of the larvae [118]. *B. subtilis* spores can withstand cooking, but are not considered a problem in food, so their addition to BSFL in future commercial insect rearing should not be a problem. Powdering the insects and heating, drying, UV treating, high-energy microwaving, pasteurizing, acidifying, or otherwise treating the meal against microbes, parasites, and bacterial spores would reduce the chance of microbial contamination relative to whole, unprocessed insects [112]. Food safety issues must be addressed before insects can enter regulated markets such as Europe [119,120,121] and the USA [94], but edible insect producers are already working on these issues [122,123]. Several reviews exist on general insect food safety issues and ways to manage these [124,125].

Concerns over heavy metal accumulation in the larvae have been partially studied. BSFL concentrate cadmium from their diet (having higher concentrations of cadmium in their bodies than their food substrate did), while concentrations of chromium, arsenic, nickel, and mercury do not surpass those of the substrate [126,127,128]. Zinc is incorporated at a decreasing rate as its concentration in the substrate increases [128]. One study claimed BSFL accumulate lead well above the concentration in the substrate [127] but another claimed they do not [128], so tests on lead should be repeated. BSFL do not seem to accumulate mycotoxins such as aflatoxin, pesticides such as chlorpyrifos, azoxystrobin, and propiconazole [126,129,130], or pharmaceuticals such as carbamazepine, roxithromycin, and trimethoprim [130], whose half-lives are shorter in the residual compost left behind than in untreated control substrates. In the case of metals, we see how the roles of BSFL as waste manager and food source conflict: removing heavy metals from wastes, where the unconverted portion is composted, would be an environmental benefit of processing wastes through BSFL, but would reduce the edibility of the larvae.

Note that, while black soldier fly larvae and pupae are safe for humans to eat, their eggs are not. Though rare, several cases of enteric/intestinal myiasis (infestation of the digestive tract by fly larvae) by BSFL exist, attributed to the consumption of fly eggs on ripe, unwashed fruit on which the flies had oviposited [131,132,133,134], as well as at least one case of cutaneous myiasis (wound infestation by fly larvae) [135]. This will not be an issue in dried, cooked, and/or powdered BSFL products, in which no viable eggs or larvae will be present. The same antimicrobial processing methods mentioned above would destroy any eggs as well.

### 2.4. Rearing Strategies for Black Soldier Fly

As mentioned in the introduction, BSFL can eat a wide range of organic materials, including animal manure, kitchen scraps, and agricultural waste. Some of these wastes are notoriously difficult to valorize, such as rice straws, which are high in lignocellulosic matter and thus are low quality as livestock feed [51,136]. Some of these wastes are potentially biohazardous pollutants and/or attract disease-vectoring pest flies, and all represent reduced revenue both in terms of lost nutrients as well as the cost of disposal. Wild BSFL are already used to manage manure successfully [48], reducing odor and pest fly populations [49]. Indeed, in parts of the world where *H. illuscens* is native and active all year, no special facilities are needed to rear large numbers of larvae. Examples include open systems allowing the flies to colonize poultry or swine manure in the farm while providing a path for wandering prepupae to follow when self-harvesting [45,70,93], or simply spreading a prepared substrate on the floor of a structure and waiting for flies to come oviposit [137].

Various low-cost rearing systems such as CORS (Conversion of Organic Refuse by Saprophages) have been developed to use biosolids such as market wastes and human feces to rear BSFL as animal feed [72,138]. An advantage of these systems is that no new facilities or buildings are required [45,48]. Pilot and full-scale plants for BSFL rearing at an industrial level have been tested and found to be effective, but technical questions still remain in scaling up the extant BSFL systems, not to mention ensuring human food quality standards [46,139,140].

A balance must also be struck between removing/recycling waste biomass and the production of the new larval biomass for food: which is the primary goal of the facility? Economically, the answer will determine whether the facility needs to compete with extant waste management systems or extant edible insect farms for efficiency [140]. Practically, the rates of biomass recycling can be altered by controlling substrate rationing rates. In an experiment with cow manure, adding greater volumes of manure daily to the BSFL led to lower development times for heavier larvae that became longer-lived adults, but the percentage reduction of manure was less [141]. Tests with human feces found longer development times and larger larvae when all food was presented at once rather than incrementally, but no effect on prepupal weight [55]. (The same study also noted that BSFL conversion rates on human feces were higher than those previously reported for swine or chicken manure or municipal organic waste, with implications for public health and sanitation, though most likely not for food production.) Some optimal feed rates have already been calculated. For ideal food waste and human feces biomass reduction abilities, daily rations of 3–5 kg/m^2^ and 6.5 kg/m^2^ respectively are recommended. Such a maintained colony could ideally produce 145 g/m^2^ dry mass of BSL prepupae daily [72].

Other aspects of BSFL rearing are being studied, which would need to be addressed before any commercial-scale rearing of BSFL as food. Elevated zinc concentrations are a potential toxicant [64]. At least one parasitoid, *Trichopria* sp. (Hymenoptera: Diapriidae), was identified to prefer BSFL over house fly larvae [76]. Temperature conditions should be as high at 30 °C to maximize development rates, but survivorship sharply decreases past this threshold to nearly no survival at 36 °C [142]. The water content of the waste must be no lower than 70% [143]. Furthermore, as the solid waste is consumed, a drainage system will be needed to remove stagnating liquid that would limit larval access to food; otherwise, the construction of the facility/bioreactor should address this issue [64]. Ultimately, however, the rearing of BSFL is considered quite easy. The greater challenges lie in rearing the adults and encouraging mating and oviposition [48], which has not been an issue in regions where wild populations are sufficient, but which must be addressed for anyone wishing to reach commercial level production year-round and in colder climates. One challenge is the space required for aerial mating. Mating and oviposition has been reliably achieved in greenhouses with 2 × 2 × 4 m screen cages [48] and 1.5 × 1.5 × 3 m nylon cages in 7 × 9 × 5 m greenhouses with sunlight and adequate space for adults to mate in the air [144], yet egg production has been recorded in cages as small as 27 × 27 × 27 cm [145]. Another challenge is sunlight, as all mating action occurs in the daytime, with the majority from 1200 to 1700 h, and preferably in a dry place [146]. Artificial light will be necessary to rear the insects in certain parts of the world and certain times of year when sufficient sunlight is not available, although finding suitable lights is a challenge. Quartz–iodine lamps [147] produced mating rates of 61% of sunlight, while no mating occurred under rare earth lamps. Supplementary light-emitting diode [148] lighting in small-scale chambers had effects equivalent to two hours of sunlight [145]. Water must be provided, but not food, although sugar supplementation of the water improves adult longevity [145].

Thanks to their self-harvesting behavior, BSFL can be separated from their substrate by simply building a path upwards in their rearing chamber that leads to a collecting container and letting the prepupae harvest themselves [45,137]. In theory, this could lend itself to automation of industrial-scale BSFL rearing and processing. Preparation of meal can be done with as little technology as sun-drying the maggots and pounding them with a hammer mill [71], which is ideal for developing world economies. Primary dehydration of BSFL will improve shelf life and serves to boost the macronutrient content: the values in Table 1 are all based on dry weight of insect meal, while protein and fat contents of BSFL “as is” are closer to 17.5% and 14%, respectively [107]. Defatting is likely the most popular subsequent processing step for BSL, to be done with mechanical pressing prior to milling. The removed fat itself may have culinary or feed supplementation uses, or use in biodiesel production. Flavor intensity can be reduced with solvent or super-critical carbon dioxide extraction [149], which can also be used for defatting [150].

The research on industrial-scale insect processing, including safety aspects [151], is limited due to the dearth of insects in the cuisines of regions with heavy food-processing industries such as Europe and the United States [125]. There are companies that specialize in commercial BSFL production as animal feed, but their methods are proprietary and, thus, unavailable to academics. This is not necessarily a problem: should demand for BSFL rise, new companies will enter the production market and invest in R&D to better compete. Innovation also need not arise from corporations, as inventors and industrial designers take up the cause of edible insects [123]. Following rearing, proper preparation, disinfection, processing, packaging, and storage throughout the process are needed to make BSFL or any other insects a safe human foodstuff for regulated markets [112]. It is possible to do, but research specifically on the preparing challenges of manure-feeding insects for human consumption has not been published at this time.

Home rearing systems are one option for edible insects, as “mini-livestock” can be reared in a far smaller space than mammals or birds [42,152]. In some rural/suburban areas, people are already rearing BSFL themselves in homemade or online-ordered containers and using food waste to attract wild flies to lay eggs, either to compost the waste and/or to rear feed for fish or livestock. Despite fears in the literature that the space required for adult mating may preclude household-sized, self-replenishing fly colonies, in recent years researchers and even private inventors have shown or at least claimed that *H. illuscens* can indeed be reared through complete life cycles on countertop containers [123,137,145]. The priorities for a home BSFL reactor are different from a farm or food factory: namely, efficient elimination of the food waste as soon as possible to reduce odor, and low labor requirements. The future economy of insects in human food likely rests not on household-sized bioreactors, however, but farm- to industrial-level systems, such that insects can be packaged and sold to food retailers. Supply of edible insects is already lacking, but supply often drives adoption of novel foods. Until BSFL and other insect products are found on supermarket [153] shelves, at least in much of the urbanized world, their human consumption will remain a niche market [14,19,154,155,156]. This problem is not unique to humans: the supply of BSFL also limits their utility as an animal feed [82].

### 2.5. Legal Regulations Regarding Black Soldier Fly

Closely tied with food safety and issues of supply is food regulation [82]. Areas without traditional histories of entomophagy and with food policies that prioritize risk avoidance, namely Europe [153,155,157,158], have more stringent rules about insects as a “novel food” that must be addressed before insects can be marketed as human food [119]. A search of FAOLEX, the United Nations’ Food and Agriculture Organization’s publically available database on food regulations worldwide, has at this point a single entry that specifically mentions black soldier fly. Dating to May 2017, the regulation [121] identifies seven insect species “currently reared in the Union”, including *Hermetia illucens,* that fulfill the safety conditions for insect production for farmed and pet animal feed. Namely: “these should not be pathogenic or have other adverse effects on plant, animal or human health; they should not be recognized as vectors of human, animal or plant pathogens and they should not be protected or defined as invasive alien species.” They also place restrictions on the substrates fed to BSFL or these other species: the substrates must contain “products of non-animal origin” or a limited set of animal products that includes fishmeal, rendered fats, blood and gelatin from non-ruminants, milk, eggs, honey, etc. Flesh is not listed, and manure, “catering waste” (human food waste), and “other waste” are explicitly excluded [121]. These restrictions eliminate the risk of prion contamination of the BSFL, but greatly limit its usage to close nutrient loops.

In the United States of America, animal feed is considered a “food” and should be regulated by the Food and Drug Administration (FDA); however, the FDA has an official Memorandum of Understanding with the Association of American Feed Control Officers (AAFCO) for all regulations regarding animal feed [94]. The FDA and AAFCO would regulate BSFL production, packaging, labeling, distribution, sale, import, and export for direct human and animal consumption respectively. In August 2016, AAFCO approved the dried larvae of *Hermetia illucens* “that has been raised on a feedstock composted exclusively of feed grade materials (and which) must contain not less than 34% crude protein and 32% fat on an as-fed basis” for use in feeding salmonid fishes [159]. At this time, therefore, BSFL cannot be reared on non-feed grade substrates or fed to non-salmonids. Rearing BSFL on chicken manure and feeding them to fish or chickens or humans is thus not allowed in the USA at this time. Requiring feed-grade substrates for BSFL greatly reduces their environmental benefit; and the protein and fat floors, which were stipulated to ensure consistent product, further limit the types of feed suitable for the larvae and, therefore, their environmental benefit [94]. The authors regrettably cannot include information on food regulations, whether they reference *H. illuscens* or not, for all other nations.

Regarding international bodies, insects are not listed in the *Codex Alimentarius*, a United Nations document on what is considered “food” that informs much global food regulatory policy, except as impurities that contaminate food [20]. This is a problem in the USA as well, where insects are described as a “defect” that can only be found in foods up to a certain point, but not explicitly stated as food [160]. Note that insects are currently sold in the USA and other nations as novelty foods for humans, with the unstated understanding that if the food is supposed to contain insects, then the insects are not a defect [16,161]. Still, the legal perception of insects as a contaminant and not a food and the general human perception of insects as unwanted in the food are both barriers to BSFL or any other insect being normalized as food [156]. Until insects are added to the *Codex* as foods rather than just impurities, they will have a significant, but not insurmountable, barrier to becoming a legally well-accepted foodstuff, regardless of what consumers accept.

### 2.6. Records of Human Consumption

Records of human consumption of *Hermetia illucens* are difficult to find [162]. Part of the problem is that ethnographers are not always entomologists, nor are the local people eating an insect likely to use its scientific name, precluding precise identification of what species one is eating [162,163]. We found only one unambiguous example of humans eating black soldier fly. In the Sabah province of Malaysia on the island of Borneo, more than 60 species of insects are eaten, predominantly by certain groups of the indigenous Kadazan-Dusun people, an ethnic group comprising almost 1/3 of Sabah’s population. BSFL are one of these, eaten raw along with a locally brewed, fermented beverage called *tapai* [164]. The larvae are collected from the fermented tapioca that is used in the making of this beverage [164]. The author noted that the Kadazan-Dusun are a primarily rural, subsistence-farming people. Most Sabahans, especially urban dwellers, see entomophagy as “disgusting, primitive, and weird” [165]. Even among the Kadazan-Dusun, entomophagy is fading: mostly the elderly practice or practiced it, with many survey respondents saying they only ate insects in their childhood [164]. Younger Sabahans have mostly abandoned the practice, and now often find it repulsive. Note too that the BSFL consumed had a food-grade substrate, and that BSFL are not the most popular insect food in Sabah. Those would be honey bee brood, grasshoppers, and the sago grub, *Rhynchophorus ferrugineus* (Coleoptera: Curculionidae), which is eaten across Southeast Asia and Oceania and is even being sold to Western audiences, to the point that it is considered overexploited [6].

Another putative record is archaeological. Fossilized early-instar BSFL were identified from coprolites (fossilized human feces) in caves in Tamaulipas, Mexico [166]. The fossils dated from 4300 to 260 years ago. It was impossible to determine the exact instar of the larvae given their condition, but they were definitively not the more distinctive, late-instar prepupae, and so were described as “early instar.” Insects in coprolites have been used as evidence of entomophagy before [167], and these Mexican findings were interpreted by Mitsuhashi in his recent compendium of edible insects as evidence of humans intentionally eating “*Helmetia* (*sic*) *illucens”* [162]. We must disagree: BSFL seem to have only ever been eaten as prepupae or late-instar larvae, never as early-instar larvae. Such small larvae would have to be manually plucked from their substrates and deliberately consumed, which seems unlikely, as most people familiar enough with black soldier flies to think to eat them would likely wait for larger larvae, and the nearly 4000 years between the oldest and youngest coprolites means plenty of time elapsed for people to discover the self-harvesting behavior. Thus, it is unlikely that the ancient Tamaulipan ate BSFL deliberately. Instead, the fossil maggots in the coprolites more likely had either hatched from eggs laid on the feces by adult flies, or, at most, hatched in the gut of the defecators due to accidental intestinal myiasis from fly eggs on another foodstuff. Mitsuhashi [162] also cites a 1997 report by Ramos-Elorduy [163] claiming the species is raised as food. The original paper on global entomophagy lists *Hermetia illucens* in a table (IX) of “edible insect species purposely raised in Mexico and other countries”, but does not indicate where or by whom the fly specifically is reared. The only information is that BSFL is *not* one of the insects “raised in a rustic way in Mexico”. Ramos-Elorduy’s other ethnographic studies of Mexican entomophagy make no references to BSFL rearing [2,168,169]. We thus cannot state that BSFL was or is consumed in Mexico or anyplace else in the world other than Sabah.

It is possible for interested consumers to purchase dried whole BSFL, BSFL oil, and defatted, milled BSFL from companies producing these for animal feed. One edible insect blogger reports that dried, whole BSFL has a pungent and unappetizing, fishy aroma (and we ourselves vouch that the live insects do not smell any better). The taste is better than the odor: an earthy, chocolate/malt flavor with far lower fish notes and a soft, melt-in-your-mouth texture. Defatting had a mild negative effect on flavor, and the oil had more pronounced chocolate/malt flavors [149]. In personal communication with these authors, other consumers report less positive opinions on BSFL flavor, though the methods of cooking likely affect BSFL flavors, as they do most other foods.

## 3. Discussion

Black soldier fly is indeed one of the nearly 2000 species of insects already consumed by humans in entomophagous cultures, but is neither popular nor common, with contemporary consumption limited to one ethnic group that is increasingly abandoning the practice [162,165]. This matches reports for insects around the world: entomophagy is primarily practiced by more rural and traditional cultures (“primitive” people) rather than urbanites, and is decreasing in popularity among these people over time as urbanization and Westernization take over [13,33,170]. *H. illuscen*’s tendency to colonize unpalatable and biohazardous wastes such as manure likely explains why it is not consumed as widely as it is geographically located, as care would be needed to clean the prepupae sufficiently. This saprophagy also means that most people’s image of BSFL, if they have any, would be as maggots that infest highly inedible dung and waste, meaning that, by the anthropological law of contagion (“once in contact, always in contact”), the larvae will always be seen as highly inedible, even if they were reared on a non-waste substrate and sterilized before cooking [171,172]. This issue affects insects in general, but for a known saprophage the effect is arguably rational, as they really might be pathogenic to eat. Research has confirmed that, for Western and non-Western consumers alike, insects associated with waste are seen as the most disgusting and least potentially edible, even if consumers know the insect dishes presented to them are safe to eat [173]. These factors, combined with the odor and taste issues associated with waste, similarly explain why the ubiquitous house fly (Muscidae) and blow fly (Calliphoridae) maggots are not typically consumed. The exception that proves the rule is the Sabah case, where the BSFL consumed are those found infesting the same food (*tapai* feedstock made of cassava or rice) with which they are eaten. By the same law of contagion, the flies are seen as similarly edible as the *tapai*. BSFL here are consuming a food-quality substrate, which normalizes their edibility (and would also allow for the legal use of these larvae as feed under U.S. law). A good parallel would be *Piophila casei* (Diptera: Piophilidae), the cheese skippers, which colonize human food as well as old human cadavers [174]. Though not reared directly for human consumption, other than another ambiguous record in Ramos-Elorduy 1997 [162,163], and never consumed off the dead, they are an example of edible entomology in the form of *casu marzu*, a Sardinian cheese fermented to the point of decomposition and colonized by these maggots, which are only ever consumed along with the cheese (a practice that skirts EU food laws) [158]. For both the cheese skipper in Sardinia and the black soldier fly in Sabah, the larvae are only ever eaten with the same food item on which they were found. The same larvae harvested off of waste would not be consumed.

Regardless, one must conclude that humans can and do eat BSFL in some situations. Whether BSFL should be an insect of choice for commercial insect rearing for human food is the main question raised by our review. Nutritionally, BSFL are acceptable for human feeding. The main concern from studies on BSFL as fish or poultry feed was reduced performance when used as the sole food source due to low palatability or nutritive content relative to other diets: many, though not all [82], studies recommended BSFL be used only as a partial meal replacement [175]. This concern is somewhat moot in humans: as omnivores, the idea of using BSFL or any other insect (or any other animal or plant organism, for that matter) as a sole source of human nutrition is not seriously considered. BSFL have one of the least healthful fatty acid profiles compared to other insects, based on our current opinions of which fatty acids are considered healthful. Interest in lowering rates of human cardiovascular disease would suggest BSFL as an unfavorable insect food relative to others due to this high saturated fat content, although one makes an assumption about future human food preferences in predicting that consumers will be choosy over which insects they eat. Defatting the insects may be essential for the commercial preparation and marketing of BSFL as a healthful alternative to animal meat, although this adds a step and cost to the processing of the insect that would need to be factored into environmental and economic analyses of BSFL rearing practicality. The importance of the substrate in determining the fatty acid profile in BSFL means some of the most desired waste biomass inputs, such as fish offal, may naturally mitigate the fatty acid problem. More data correlating substrate type to nutrients in the larvae are needed. From a sensory perspective alone, studies have found that lower (more healthful) ratios of monounsaturated to polyunsaturated fats beyond a certain threshold are associated with negative odor, texture, and flavor profiles [176,177,178,179]. Thus, though perhaps less nutritionally favorable, BSFL might be tastier than other insects. The high levels of saturated fats in mammal meat, poultry skin, and dairy products have not stopped these foods from being accepted in human diets. Note also that the high amounts of medium-chain saturated fatty acids and low amounts of polyunsaturated fatty acids found in BSFL, while not ideal for human health, are ideal for biodiesel fuel production, suggesting that BSFL reared on waste may be even better suited as fuel than food [100]. Research on commercial-scale biofuel production from waste-fed saprophagous insects and its economic and environmental sustainability are a worthy topic for further exploration.

Regarding flavor and mouthfeel, the texture of BSFL is not significantly different from other larvae, but at this time no sensory profile tests have been done on BSFL-derived food. The issues of flavor and texture do not get the attention they deserve in entomophagy texts: the assumption that insects will be accepted as meat substitutes once the taboos of eating insects are overcome ignores the problem that insects do not taste or feel like meat [156,173,180]. BSFL in particular are most frequently compared to fish meal and oil, albeit with an earthier flavor. Note that one need not always think of what extant foods a novel food tastes like, and instead should consider that an insect tastes like an insect and prepare/market it accordingly [156]. The texture issue is more prominent in insects with hard exoskeletons like crickets, but appears to be less important in the soft-skinned BSFL. Fish meal is not a major ingredient in human cuisine, but pureed fish is used to make fish balls in East Asia and Scandinavia, and such *surimi*-type products (“textured insect protein”) might indeed be a successful way to prepare BSFL or other insects for human consumers, assuming the flavor is acceptable. The advantage this formulation has is that it hides the insect origin. Many have reported, to the point of it becoming a consensus, that the best way to convince people in cultures unused to entomophagy to add insects into their diet is to disguise the insect as much as possible, with grinding the insects to a powder being the ideal method [14,15,181]. Powdered/ground insects can be easily added to baked goods and processed meats [3,182]. This should also work to introduce a typically unappetizing insect to people who eat other insects readily, as different entomophagous cultures are not united in their food choices, but see different types of insects as delicacies or disgusting [25]. People surveyed also seem to prefer meat products that are part-insect, part-conventional livestock meats as opposed to all insect or all vegetable meat alternatives [183]. Insect meal has various uses, such as fortifying baked goods for extra protein (a ratio as high as 50/50 grain: insect meal has the same baking performance as pure flour), formulation into protein bars, or use in ground animal meat substitutes [184]. More creative ways to process insects are currently being researched, which can shape the direction of the industry greatly [33,182]. For the more adventurous, dried BSFL could be a snack item suitable for eating like peanuts and salted crackers as an accompaniment to beer or other light alcohols, like fried grasshoppers in Mexico (*chapulines)* [185] or, indeed, like raw BSFL in Sabah [164]. This use of insects as a snack rather than a vertebrate protein substitute for main courses does not meet any environmentalist goals, however, but just adds insects to the diet for novelty without any nobler reason [156]; not that there is anything wrong with that [186].

Under the paradigm of insects as an alternative protein to mitigate the effects of climate change or rising populations, *H. illuscen*’s advantages might reasonably put it as the insect of choice among the pantheon of 1 million species: not only does it not compete with humans or livestock for food, have specialized diets, or require significant environmental manipulation [187], but also it provides an economic and ecological service in processing the waste substrates that it feeds on. No other insect comes close to closing so many material flow loops and creating nearly self-sustaining food production cycles as BSFL, effectively making BSFL rearing on wastes a self-financing form of pollution reduction [188,189], although it is not yet known what are the best ratios of insects to substrate and what conditions will provide the optimal mix of larval production and waste recycling. BSFL thus show strong promise as part of a sustainable system with hydroponics or composting and aquaculture or poultry farming [93,190,191], particularly in situations where food choices would be extremely limited, such as space travel [190,192] or the most pessimistic global food insecurity crises envisioned by population growth extrapolation [30]. Pilot projects to this end are already underway, such as the “Insect-based Feed and Fertilizer Production” project co-run by the Swiss Research Institute of Organic Agriculture and the University of Ghana [191]. However, the present legal restrictions on using waste-fed BSFL as livestock feed in the USA and EU mean BSFL cannot presently be legally used to close nutrient loops, but rather to recycle the nutrients from wastes into another form, such as compost or biodiesel. BSFL’s use as feed or food hinges on changes in these regulations, which in turn depend on positive results to safety testing.

Saprophagy has economic and ecological advantages but a cultural disadvantage: the idea of eating an insect that feeds on waste, like a cockroach or blowfly, simply comes with too much of a taboo [173]. Beyond preconceived expectations of bad flavors, people may still feel consuming insects that fed on animal biosolids is hazardous to one’s health, and the extant laws reflect these concerns. Though the flies have a strong record of reducing microbial and chemical contamination from their substrates, and microbial issues can be dealt with during insect production and processing [124], the possibility of something unpleasant remaining in their gut between self-harvest and final processing exists. While manure is not considered a reservoir for prions that cause diseases such as bovine spongiform encephalitis or scrapie, there is currently no data on the fate of prions in slaughterhouse wastes processed by BSFL [193]. Insects are definitively not biological vectors or amplifiers of prions [114,194,195], but experiments with the corpse fly *Sarcophaga carnaria* fed brains from scrapie-infected hamsters found that healthy hamsters can become infected after eating these fly larvae [195]. Evidence of absence would be more comforting to consumers and regulatory agents than absence of evidence, so this should be a priority research topic before BSFL fed slaughterhouse biomass can be legally fed to other organisms. Pending such data, the European Food Safety Authority recommends insects used as animal or human feed not be fed manure or certain ruminant meat substrates, some of which are already excluded from the food chain due to their risk of transmitting prion diseases. They also note that thermal treatment can deactivate prions as well [114].

Beyond manure and feces, reports exist of BSFL colonizing human cadavers in advanced stages of decay [196,197,198,199], and pig cadavers as early as a week [200]. It is hard enough to convince people to eat grasshoppers, which, as entomophagy promoters love to argue, are farm-raised and eat the same food as cows do. To eat an insect whose diet includes garbage, dung, and even the dead may be too difficult a pill to swallow, even though other saprophagous arthropods—crabs and lobsters—are readily consumed. Insect foods, including BSFL, are already associated with rurality and primitiveness [33,164]. The risk is that BSFL, perhaps more than insects in general, will be labeled a “starvation food” [201] or “food of attrition” [156]: something not eaten for its own organoleptic properties, but because humanity has no alternative. Global warming and overpopulation are thus depicted as societal crimes whose punishment is reducing humanity to eating things that most people would otherwise find objectionable [202]. Unfortunately, this pessimistic view is exactly how BSFL and other insects have been primarily marketed for human consumption in the West: with a focus on their sustainable production rather than flavor or cost, which are the greater factors driving what people choose to eat [14,154,186].

Such marketing of insects as sustainable is itself problematic, as other protein alternatives to livestock exist that are even more sustainable. This includes soy and other vegan protein sources, as well as future foods such as algae and lab-reared meat [17,203]. Plant proteins have a lower carbon footprint than BSFL and are better for human cardiovascular health due to their superior fatty acid profiles. However, BSFL have an edge over plant proteins in terms of the greatly reduced land and water requirements for their rearing, as well as their ability to recycle wastes. BSFL are no substitute for plants sensu lato in the human diet, but per gram protein may prove to be more sustainable to produce than plant protein due to the low input costs. Of all extant protein production industries (other than hunting and fishing), BSFL rearing could prove to be the most sustainable. It absolutely has a place in the future, both as a waste recycler and as animal feed. The biggest obstacle may be a regulatory/legal one: extant laws on BSFL as animal feed explicitly forbid the use of “wastes” as substrate for BSFL feeding, which immediately places limitations on BSFL rearing. Luckily, these laws (themselves less than two years old at the time of this paper’s publication) can and do change following the efforts of lobbyists armed with new research results. The biggest such research questions remaining are just how much protein and fat is produced per kilogram waste fed into a BSFL bioreactor per insect, how can BSFL be safely decontaminated if reared on a waste substrate, and whether their fatty acid profiles or palatability issues compromise their economic value as a feed substitute.

## 4. Conclusions

BSFL are edible, nutritious (especially when defatted), and can theoretically be reared more sustainably than extant farmed insects (and, therefore, extant farmed animals) pending further development of large-scale biorefineries. This makes them a potential protein source for humans both in the future and in the developing world. However, their primary usage in human food systems will likely be to reduce and recycle the waste biomass associated with other foods’ production and consumption, from farm to table to toilet, possibly followed by ancillary use as animal feed or biodiesel feedstock. Legal prohibitions already limit such waste-fed BSFL’s use as animal feed, let alone as a human food, and these will not change without favorable results of safety assessments. Other challenges in making BSFL palatable for humans are the same as those facing all insects: sensory and cultural. Processing BSFL into human food is possible, but there is no guarantee that this is the best solution to food insecurity or that BSFL can ever shed the stigma associated with being saprophagous.

## Figures and Tables

**Table 1:** Mean % crude protein (not chitin-corrected) and % fat (ether extract) per dry weight of BSFL meal, using mean values from the studies listed.

Diet or Source	% Protein	% Fat	Source
Cattle blood and wheat bran	47.6	25.3	Aniebo et al. 2009 [71]
Poultry manure	37.9	18.73	Arango Gutiérrez et al. 2004 [103]
Proprietary (Hermetia Futtermittel GbR, Baruth, Germany)	31.7	21.1	Bußler et al. 2016 [102]
Proprietary (Hermetia Futtermittel GbR, Baruth, Germany)	36.9	34.3	de Marco et al. 2015 [104]
UFA 625 chicken feed	37.86	-	Diener et al. 2009 [128]
Proprietary (Hermetia Futtermittel GbR, Baruth, Germany)	47.6	11.8	Kroeckel et al. 2012 [65]
Municipal organic waste	39.8	30.1	Mutafela 2015 [137]
Horse manure	40.9	12.9	Mutafela 2015 [137]
Fresh fruit waste	37.8	41.7	Mutafela 2015 [137]
Swine manure	43.2	28	Newton et al. 2005 [89]
Poultry manure	42.1	34.8	Newton et al. 2005 [89]
Wild (Bondo area, west Kenya)	40	33	Nyakeri et al. 2017 [93]
Food manufacturing by-product mixes	38–46	21–35	Oonincx et al. 2015 [32]
Laying hens' manure	42	35	Sheppard et al. 1994 [45]
TOTAL 77 Chicken feed	41.2	33.6	Spranghers et al. 2017b [97]
Biogas digestate	42.2	21.8	Spranghers et al. 2017b [97]
Vegetable waste	39.9	37.1	Spranghers et al. 2017b [97]
Restaurant waste (vegan)	43.1	38.6	Spranghers et al. 2017b [97]
Cow manure	-	21.42	St-Hilaire et al. 2007a [67]
50/50 Fish offal: Cow manure	-	30.44	St-Hilaire et al. 2007a [67]
Swine manure	43.2	33.1	St-Hilaire et al. 2007a [67]
Animal manures	42–44	31–35	Yu et al. 2009 [50]
